# *PGG*.SNV: understanding the evolutionary and medical implications of human single nucleotide variations in diverse populations

**DOI:** 10.1186/s13059-019-1838-5

**Published:** 2019-10-22

**Authors:** Chao Zhang, Yang Gao, Zhilin Ning, Yan Lu, Xiaoxi Zhang, Jiaojiao Liu, Bo Xie, Zhe Xue, Xiaoji Wang, Kai Yuan, Xueling Ge, Yuwen Pan, Chang Liu, Lei Tian, Yuchen Wang, Dongsheng Lu, Boon-Peng Hoh, Shuhua Xu

**Affiliations:** 1 Chinese Academy of Sciences (CAS) Key Laboratory of Computational Biology, Max Planck Independent Research Group on Population Genomics, CAS-MPG Partner Institute for Computational Biology (PICB), Shanghai Institute of Nutrition and Health, Shanghai Institutes for Biological Sciences, University of Chinese Academy of Sciences, CAS, Shanghai, 200031 China; 2 Present Address: Department of Genetics, Perelman School of Medicine, University of Pennsylvania, Philadelphia, PA 19104 USA; 3 School of Life Science and Technology, ShanghaiTech University, Shanghai, 201210 China; 4 Faculty of Medicine and Health Sciences, UCSI University, Jalan Menara Gading, Taman Connaught, Cheras, 56000 Kuala Lumpur, Malaysia; 5 Center for Excellence in Animal Evolution and Genetics, Chinese Academy of Sciences, Kunming, 650223 China; 6 Collaborative Innovation Center of Genetics and Development, Shanghai, 200438 China

**Keywords:** Human diversity, Population genetics and genomics, Single nucleotide variations, Indigenous populations, Population prevalence, Variant annotation, Evolutionary conservation, Natural selection, Disease risk allele

## Abstract

Despite the tremendous growth of the DNA sequencing data in the last decade, our understanding of the human genome is still in its infancy. To understand the implications of genetic variants in the light of population genetics and molecular evolution, we developed a database, *PGG*.SNV (https://www.pggsnv.org), which gives much higher weight to previously under-investigated indigenous populations in Asia. *PGG*.SNV archives 265 million SNVs across 220,147 present-day genomes and 1018 ancient genomes, including 1009 newly sequenced genomes, representing 977 global populations. Moreover, estimation of population genetic diversity and evolutionary parameters is available in *PGG*.SNV, a unique feature compared with other databases.

## Background

The past two decades have witnessed the exponential increase in the number of human genomic sequences [1–6] generated with genotyping or next-generation sequencing (NGS) technologies, which allow researchers to delineate the functional consequences of each variant, the fundamental goal of human genetics. Generally, there have been three major strategies for accomplishing the goal: genetic approaches, experimental approaches, and evolutionary approaches [7]. Genetic approaches such as linkage analysis and genome-wide association studies (GWASs) can identify candidate variants, but usually have insufficient power to pinpoint causal variants [7], mainly due to the linkage disequilibrium between variants located closely on an individual chromosome and the lesser power of GWAS to dissect rare variants [8, 9]. Traditional experimental methods or molecular biology techniques are generally performed to support a limited number of candidate causal variants identified for a given phenotype and are challenging to implement in humans. Now it is feasible to carry out larger-scale experimental assessment of genetic variants [10–12] due to rapid development of high-throughput sequencing technologies, which definitely have facilitated our understanding of the functional elements/variants in humans. However, some of the experimental methods used are still controversial for the determination of genomic function. For example, the biochemically active regions detected by the ENDODE project (e.g., H3K4me3 containing regions) cover a much larger fraction of the genome than do evolutionarily conserved regions, raising the question of whether the non-conserved but biochemically active regions are truly functional [13]. Though some recent experimental methods such as massively parallel report assay [14, 15] were successful in identifying expression-modulating variants, they are not ready to be applied in diverse human populations.

Compared with other methods, the evolutionary approaches that facilitate the study of the genetic legacy left in human genomes are relatively cost-effective and powerful for narrowing down candidate functional regions. The underlying rational is that the nature as a super laboratory performs functional experiments by inducing mutagenesis cross human genomes and simulating diverse conditions along the evolutionary time; regions/variants that are evolutionarily conserved or under positive selection are assumed to be functional. Up to date, substantial constraint-based algorithms have been developed to measure the deleteriousness of both protein-coding variants [16, 17] and non-coding regions [18, 19], and numerous methods have emerged and applied to larger empirical data for detecting positively selected regions [20–24]. For instance, a deleterious missense variant (rs80356779) located in the gene *CPT1A* (MIM: 600528) [25], a functional variant (rs7330796) located in the gene *TBC1D4* (MIM: 612465) [26], and several variants in proteins that metabolizes omega-3 polyunsaturated fatty acids [27] occur at high frequency in Arctic human populations and might adapt humans to either specific diets or a cold environment. Other examples include the missense variant (rs186996510) in the gene *EGLN1* (MIM: 606425), some regulatory variants of the gene *EPAS1* (MIM: 603349), and a novel missense variant in the gene *ALDH3A1* (MIM: 100660), which are candidates for high-altitude adaptation in either Tibetans [28–33] or the Sherpa people [34]. All of these suggest that evolutionary approaches have provided new insights into the functional effects of genetic variants associated with specific environments [35].

Moreover, by leveraging the laws of intra-species micro-evolution, analysis of the population prevalence of variants has increased dramatically in medical studies and functional genomics [36, 37]. Specifically, researchers are able to retrieve the allele frequency of a variant and predict the impact of that variant according to its rareness, as deleterious alleles are generally assumed to show lower frequencies in a population than benign alleles [38]. The two most-frequently used data sets for this are the 1000 Genomes Project (1KGP) [4] and the Genome Aggregation Database (gnomAD) [39]. However, both data sets are insufficient to cover the majority of ethnic groups. For instance, the 1000 Genomes Projects does not sufficiently cover the human genetic diversity in Asia [40]. Nearly half of the genomes in gnomAD are from European ancestry and merely 9% of the genomes are of African ancestry (though with the highest genetic diversity), implying a severe ancestral bias problem in human variant sequencing efforts [41]. Moreover, samples in gnomAD were merely divided into 15 groups majorly on the continental level, leaving the majority of the specific ethnic groups unknown. For example, gnomAD exomes grouped East Asians roughly into three categories: “Korean,” “Japanese,” and “other East Asians”; therefore, researchers fail to query the allele frequencies for most of East Asian populations, such as the Han Chinese, Tibetan, and Uyghur populations. In this case, researchers may inadvertently neglect variants with high disease-associated allele frequencies (DAAF) in their studied populations, as the large number of un-grouped genomes in gnomAD would dilute the DAAF, while the value would actually be higher if specific populations were investigated. The above reveals the necessity for comprehensively analyzing prevalence of variants in diverse ethnic groups between which health disparities of certain diseases probably exists. Fortunately, tremendous efforts provide us informative reference data sets for examining the genomic diversity in human populations (see Table 1). However, to the best of our knowledge, few databases archive genetic variants covering as many as ethnic groups from multiple data sets to reduce the ancestral bias.

**Table 1:** Summary of data sets included in *PGG*.SNV

Data set	Abbr. Data set	Type	Ancient (Archaic)	No. Genomes	No. Populations (Ancestries)	References
NHLBI Exome Sequencing Project	ESP	EGS	N	6503	2	[3]
Genome of the Netherlands	GoNL	WGS	N	769	1	[42]
Whole-genome sequences of 3554 healthy Japanese individuals	3.5KJPN	WGS	N	3554	1	[43]
Genome Aggregation Database	gnomAD.genomes and gnomAD.exomes	WGS and EGS	N	141,456	25	[39]
1000 genomes project phase 3	1KG_phase3	WGS	N	2504	26	[4]
Estonian Biocentre data set	Estonian_Biocentre	Genotyping	N	1297	123	[44–53]
Human Origins data set	HuOrigin	Genotyping	N	2327	202	[54]
Simons Genome Diversity Project	SGDP	WGS	N	261	128	[6]
Human Genome Diversity Project	HGDP	Genotyping	N	937	53	[2]
International HapMap Project	HapMap	Genotyping	N	1397	11	[1]
Asian Genome Diversity Project	AGDP	Genotyping	N	3605	40	[55]
Negrito Pygmy data set	NegPyg	Genotyping	N	1233	61	[56–61]
Pan-Asian SNP Consortium	PASNP	Genotyping	N	1691	71	[62]
Asian Admixed Genomes Consortium	AAGC	WGS	N	1009	16	–
Han Chinese Genomes Project	HanGenomesProject	Genotyping	N	51,094	38	–
Singapore Genome Variation Project	SGVP	WGS	N	132	2	[63, 64]
Indian genomes	Indian Genomes	Genotyping	N	378	52	[65]
The complete genome sequence of a Neanderthal from the Altai Mountains	AltaiNea	WGS	Y	1	1	[66]
A High-Coverage Genome Sequence from an Archaic Denisovan Individual	DenisovaPinky	WGS	Y	1	1	[67]
Genome sequence of a 45,000-year-old modern human from western Siberia.	Ust_Ishim	WGS	Y	1	1	[68]
Ancient anatomically modern humans	AAMH	GWADD	Y	989	109	[69–75]
Ancient anatomically modern humans (Southeast Asians)	AAMH.SoutheastAsia	WGS	Y	26	13	[76]

Ancient individuals from the same country and same age period are grouped into one ancient population. *N* no, *Y* yes, *EGS* exome genome sequencing, *WGS* whole-genome sequencing, *GWADD* genome-wide ancient DNA dataset, *No.* number of, *Abbr* abbreviation; “–”, unpublished

Compared with living anatomically modern human (AMH) genomes mentioned above, ancient genomes (including archaic hominins and ancient AMH genomes) provide more direct evidence of past human adaptation and even high-resolution snapshots of the adaptive histories of phenotypes [77–79]. However, analyzing the detailed time series of allele frequency trajectories from ancient genomes is usually ignored in many medical or genomics studies, partly due to the relatively slower development of ancient DNA sequencing technologies. Recently, more than 1000 archaic hominin and AMH genomic sequences are now available, covering time periods from 430,000 years before present day to the early twentieth century [78]. Systematically leveraging these data may therefore facilitate an understanding of how genetic variants evolve in response to new environments and how adaptation impacts on health and medicine today [78].

The current substantial number of human genomes and comprehensive catalogue of genetic variants available provide researchers with an extraordinary resource for dissecting the evolutionary and medical implications of human single nucleotide variants (SNVs) at a population level. To realize this, we first sequenced more than 1000 genomes from East Asia and South Asian to 10–30× coverage, and collected publicly accessible data sets and integrated the two. We built a user-friendly database (*PGG*.SNV, https://www.pggsnv.org for genome build GRCh37 and https://grch38.pggsnv.org/ for GRCh38), which documents 265 million SNVs, featuring more than 10 billion allele frequency records, for 220,147 present-day human genomes and 1018 ancient genomes from 977 populations. Based on the database, we then investigated the characteristics of Mendelian-inherited disease-associated alleles (DAAs) to address the following scientific questions: (1) what is the allele frequency spectrum of DAAs according to *PGG*.SNV; (2) which DAAs distribute disparately between populations/ancestries; and (3) which groups harbor heavy genetic loads for specific diseases. We suggest a helpful population prevalence analysis as a reference procedure for predicting and prioritizing causal variants for Mendelian-inherited diseases.

## Construction and content

### Data generation and collection

To improve studies of the genetic diversity of humans, we generated or collected genomic data from different human populations (Table 1 and Additional file 1: Table S1). The newly generated whole-genome sequencing data (1009 genomes from 16 ethnic groups in Asia) were sequenced by the Asian Admixed Genomes Consortium (AAGC). Meanwhile, we worked together with our collaborators as well as other initiatives in the Asia-Pacific region, and sequenced or genotyped the genetic variants of diverse East Asian and Southeast Asian populations. We also collected publicly accessible genomic data sets that covered not only general populations studied by international projects, such as the HapMap Project [1], the Human Genome Diversity Project [2], the 1000 Genomes Project [4], the HUGO Pan-Asia SNP Project [62], the Human Origin data set [54], and the Simons Genomic Diversity Project [6], but also genomic data sets from indigenous or isolated populations that were contributed by regional sequencing efforts, such as the Singapore Genome Variation Project [63, 64], and genomic data sets from ethnic groups with genomes deposited in the Estonian Biocentre (Table 1 and Additional file 1: Table S1). The aforementioned data represent the great genomic diversity of the human population as described in the *PGG*.SNV associated database, *PGG*.Population [80]. Besides genomic data, allele frequency data were also collected from data sets with a substantial number of samples, such as the Genome Aggregation Database (gnomAD) [39] and the NHLBI Exome Sequencing Project (ESP) [3], as well as frequency data from 3554 healthy Japanese individuals [43]. Although there could be some overlaps, for example, the frequency information of ESP is already included in gnomAD, we treated these datasets as independent so that the sources are traceable. Beside present-day genomes, we collected many ancient human and archaic hominin genomes (ancient genomes hereafter), of which the ages ranged from 430,000 years before present day to the early twentieth century, covering the landscape of genomic diversity across the human evolutionary time scale. All data sets and populations included in the database are summarized in Table 1 and Additional file 1: Table S1, respectively.

### Data integration, quality control, and upstream analysis

**Fig. 1:**
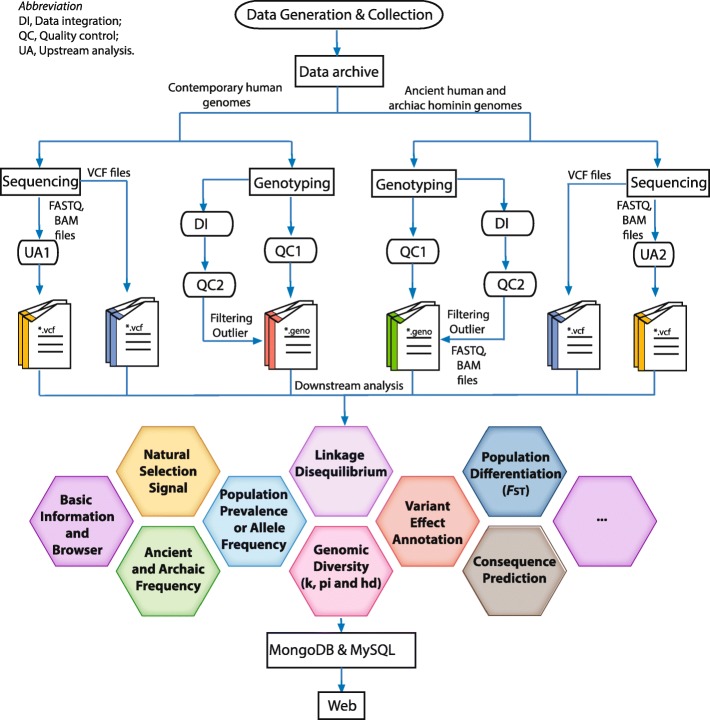
Analysis framework for data generation, collection, integration, and annotation. The ellipsis in the right hexagonal represents other population genomics analyses that are not included in the current version of the database but would be performed in later versions

Different data analysis processes were performed based on the type of the genomic data (contemporary vs. ancient genomes), as well as the data type (sequencing or genotyping data) (Fig. 1).

For sequencing data from contemporary genomes with raw data, we analyzed each set from cleaned fastq files (Additional file 2: Supplemental methods). Short reads were mapped to the human reference genome (GRCh37) using “mem” algorithm “bwa mem –M –R @RG\tID:name\tSM:name” in the Burrows-Wheeler Algorithm (BWA) [81]. Duplicated reads were removed using Picard. Base quality score recalibration (BQSR), single nucleotide variant (SNV) calling, and variant quality score recalibration (VQSR) were carried out using the BQSR module, the HaplotypeCaller module, and the VQSR module in GATK [82, 83], respectively. Variants in joint VCF file with phred-scaled quality score less than 30 (QUAL < 30) were considered as low quality and therefore were filtered out. Variants within complex regions in the human reference genome where the variant calling can be challenging were also removed following the pipeline described elsewhere [6]. The methodology details for the raw sequencing data processing were summarized in the Additional files. For 3.5KJPN and gnomAD data sets with only VCF files, only variants labeled “PASS” in the QUAL column of VCF files were retained. For ancient genomes with raw data, we used BAM files for upstream analysis, as previous studies were assumed to have carried out strict quality control for ancient short reads. The variant calling and filtering approaches were similar to the strategies used for contemporary genomes as mentioned above.

We controlled the quality of each genotyping data set at two levels. First, within data sets, we removed SNVs with a call rate of < 90% (across all individuals) and required at least 90% genotyping completeness for each individual (across all of the SNPs). We also discarded recently related individuals by filtering one individual from all of the pairs when identity by descent (IBD) was > 0.25. Please note that this IBD threshold only removed the second degree of the relatedness; some indigenous ethnic groups of small population size could retain some samples with third-degree relatedness, although most populations are not affected. Second, we integrated each data set into the 1KGP data to estimate the data quality by performing principal component analysis (PCA) (Additional file 2: Figure S1). Outliers were identified using the *PGG*.Population web tool [80] and were then removed from genotyping data sets. At both levels, strand information was determined from the whole-genome sequence data based on the Human Genome Build 37 positions, and a strand was flipped to match that of the sequenced data. At both levels, all of the A/T and G/C markers were removed to reduce the risk of any ambiguity.

To document SNVs with both genome assemblies, we converted the coordinates of all dataset, except for 1KGP, from GRCh37 to GRCh38 with Picard. For 1KGP, we directly obtained the VCF with GRCh38 assembly from the official website. Data sets for both genome builds were further applied for annotation and other downstream analysis.

### Population and ancestry assignment

In the context of *PGG*.SNV, population or ethnic group refers to a kind of “inherited” status of shared genetic ancestry, language, history, society, culture, or nation. For present-day human samples, populations were firstly verified based on PCA (Additional file 2: Figure S1). Population and/or sample outliers that are in conflict with the geographic origin of sampling and/or self-reporting (reported by each data set) would be excluded in our database. Populations with extremely large sample size and clear sampling locations were divided into different subgroups. The Han Chinese from Han Chinese Genomes Project (*n* = 51,094) is the only case in the current version of *PGG*.SNV, as it is the world’s largest ethnic group and previous studies have shown their sub-structures [84–86]. Each population was further assigned into the following eight geographical groups with ancestries derived from the continent where the group is residing: African, American, Central Asian and Siberian, East Asian, Oceanian, South Asian, Southeast Asian, and West Eurasian. For ancient human genomes, we assigned populations based on geography and their time periods, as we do not know exactly which ethnic group they belong to. The time- and geography-based population assignment for ancient genomes facilitate us to trace the allele frequency fluctuation through history and thus to understand the genetic origin of a specific variant. All populations and their ancestry information can be obtained from Table S1 and the user guide section on the *PGG*.SNV website.

### Variant annotation and other downstream analysis

Variant effect and conservation scores were performed using a variant effect predictor [87]. The population prevalence of variant for each population was calculated from the genotype counts of the corresponding population. The population differentiation measured by *F*_ST_ between each pair of populations was calculated following Weir and Cockerham [88]. Natural selection was analyzed using SelScan [89]. Genomic diversity and linkage disequilibrium were calculated in real-time using VCFtools [90]. For sequencing data set without available genotypes on an individual level, such as the 3.5KJPN and gnomAD data sets, analyses of natural selection, genomic diversity, and linkage disequilibrium cannot be performed by *PGG*.SNV.

### Analysis of population prevalence for Mendelian-inherited disease variants

The variants associated with Mendelian disorders were obtained from ClinVar, where variants have been grouped into five categories ordered by the severity of disease: (1) pathogenic, (2) likely pathogenic, (3) uncertain significance, (4) likely benign, and (5) benign, according to the recommendation of the American College of Medical Genetics and Genomics and the Association for Molecular Pathology (ACMG/AMP) [91].

The alternative allele frequencies for all of the Mendelian-inherited disease variants were calculated over all populations with sample size larger than 5 of each data set, each ancestry, and our entire database. To estimate the frequency differentiation between populations or ancestries, we used the formula _*z*_$\mathrm{Diff}=\max \left(\mathrm{A}{\mathrm{F}}_i\right)-\overline{\mathrm{AF}}$, where AF_*i*_ represents the alternative allele frequency of the *i*th population/ancestry, and $\overline{\mathrm{AF}}$ is the mean frequencies of all of the populations/ancestries, calculated from the formula $\overline{\mathrm{AF}}=\frac{\sum_{i=1}^n\mathrm{A}{\mathrm{F}}_i}{n}$. Since we focus on Mendelian disease variants which may have severe effects than other variants, they are in relatively low frequency in human and are usually not highly differentiated among populations. We examined the distribution of the allele frequency of disease variants in the *PGG*.SNV database and observed that the top 5% of the frequency is around 0.1. We therefore defined variants with Diff_pop._ > 0.1 as variants that largely differ between populations and variants with Diff_Ances._ > 0.1 as variants that largely differ between ancestries. Largely differentiated variants between populations/ancestries were sorted by relative difference (RD), which was defined as $\mathrm{RD}=\frac{\mathrm{Diff}}{\mathrm{AF}}$.

### Website design and database back-end

*PGG*.SNV is available at https://www.pggsnv.org and requires no username or password. The static web technology used included HTML5, CSS, and the Bootstrap framework. To enhance the user experience, JavaScript, jQuery, and ECharts were implemented. The dynamic web was built using Java and a Spring MVC framework. Integrative genomic viewer (IGV) [92] was embedded into the web to allow the visualization of variants. Genomic data were stored using a Huawei data storage system. Annotation data were imported into MySQL and MongDB. The data on natural selection signals were JSON-formatted, so data could be recognized and plotted by LocusZoom.js in the front webpage. We receive email inquiries and give timely responses at pggadmin@picb.ac.cn, and any suggestions on the website and database are welcome.

## Utility and discussion

### Comprehensive genetic diversity and variant annotation in diverse populations

**Fig. 2:**
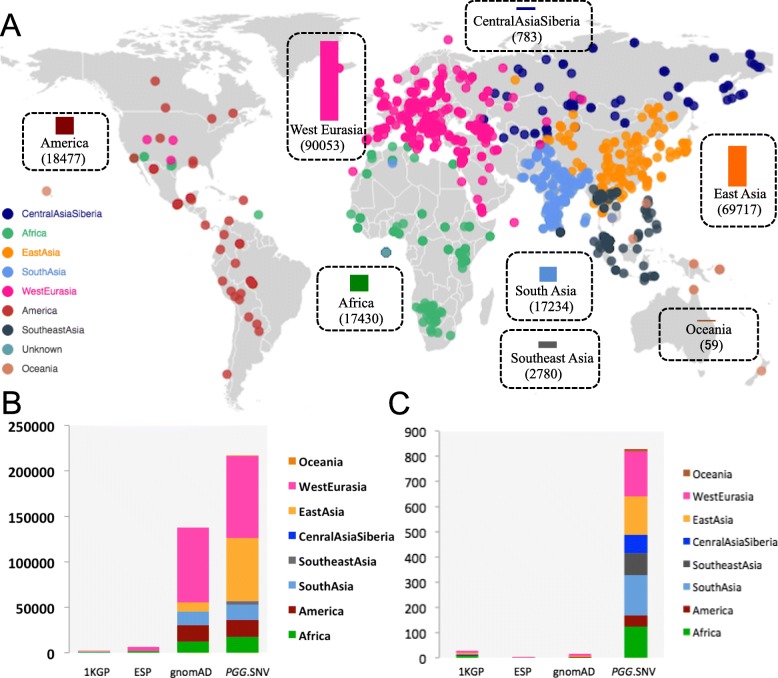
Comparison of the number of genomes and populations between *PGG*.SNV and other frequently used data sets. **a** Geographical distributions of the population samples included in *PGG*.SNV. Each dot represents an ethnic group, and each bar denotes the number of genomes of the corresponding ancestry. **b** A comparison of the numbers of genomes included in the 1000 Genomes Project (1KGP), Exome Sequencing Project (ESP), The Genome Aggregation Database (gnomAD), and *PGG*.SNV. Each color represents an ancestry that was used in **a**. **c** A comparison of the numbers of populations or ethnic groups included in different databases. *PGG*.SNV includes 852 present-day populations and 125 ancient populations which is defined based on geography and time period

*PGG*.SNV currently consists of 220,147 modern human genomes comprising different genetic ancestries (African [*n* = 17,430], American [*n* = 18,477], Central Asian and Siberian [*n* = 783], East Asian [*n* = 69,717], Oceanic [*n* = 59], South Asian [n = 17,234], Southeast Asian [*n* = 2780], West Eurasian [*n* = 90,053], and Unknown [*n* = 3617]) from 852 distinct present-day ethnic groups (African [*n* = 130], American [*n* = 47], Central Asian and Siberian [*n* = 70], East Asian [*n* = 159], Oceanic [*n* = 11], South Asian [*n* = 163], Southeast Asian [*n* = 89], West Eurasian [*n* = 181], and Unknown [n = 2]) (Fig. 2a).

Compared to other frequently used data sets, *PGG*.SNV documents more genomes (Fig. 2b) and represents a much more comprehensive genomic diversity of worldwide populations (Fig. 2c). For instance, there are 90,514 Asian genomes included in *PGG*.SNV, compared to 993 and 25,285 in the 1KGP and gnomAD data sets, respectively. Remarkably, our database integrate hundreds of populations from diverse data sets, while each data set alone merely covers a small number of ethnic groups (e.g., 1KGP) and some data sets such as gnomAD assign genomes majorly based on continent, leaving the specific information for populations ambiguous (Fig. 2c). Moreover, *PGG*.SNV includes 1009 newly generated whole-genome sequences from 16 ethnic groups, especially many indigenous groups living in East Asia and Southeast Asia whose genomes have not been sequenced before (Additional file 1: Table S1). Besides present-day human populations, the database integrates 1018 ancient genomes (including two archaic hominins and 1016 ancient AMHs) that represent time periods from the 430,000 years before the present day up to the early twentieth century, which, to the best of our knowledge, is rarely considered in many other existing databases.

The genomic data from numerous populations with different ancestries represent a comprehensive catalogue of human genetic variation, comprised of 265 million SNVs as of March 2019. We therefore annotated each variant based on numerous aspects including, but not limited to (1) basic information and variant browser, (2) population prevalence or allele frequency, (3) ancient and archaic frequency, (4) variant effect annotation, (5) consequence prediction, (6) population differentiation, (7) natural selection signal, (8) genomic diversity, and (9) linkage disequilibrium (LD) (Table 2 and Fig. 1). Each type of annotation aimed to dissect the evolutionary and medical implications of human single nucleotide variants at the population level. Annotations (1) and (4) offer information such as the genomic location, variant type, and gene content, for each variant. Annotation (2) provides the population prevalence of variants in contemporary populations, which enables studies of variants that are rare or absent in many well-studied populations, further guiding Mendelian-inherited disease mapping studies. Annotation (3) provides the population prevalence of variants in ancient groups, facilitating an understanding of the evolutionary trajectory of genetic variants as well as the gene flow or potential introgression events. Annotations (6), (7), and (8) enable the detection of the genetic legacy (within species) left in human genomes, as these regions or variants have been assumed to be functionally relevant. Annotation (5) uses different algorithms such as CADD [18] and GERP [19], predominantly based on conservation information between species, to predict the functional consequences of each variant. Annotation (9) provides the genetic linkage between a given variant and its surrounding loci, which may improve the interpretation of phenotype-genotype association studies.

**Table 2:** Summary of variant annotation types and their web illustration elements

No.	Annotation	Description	Illustration
0	Basic information	Basic information, such as allele status, alternative allele frequency of all genomes as a whole, and variant-related links	Card
1	Variant browser	The Integrative Genomics Viewer (IGV) to visualize genomic data sets, such as human and ancient reference genomes, and conservation scores	IGV browser
2	Population prevalence	Alternative allele frequency (AAF) in worldwide populations	AAF distribution map; Table
3	Ancient frequency	Alternative allele frequency for selected variant in ancient genomes	AAF distribution map; Table.
4	Variant effect	Variant types, effects, and gene contents for selected variant	Table
5	Consequence prediction	Consequence predicted Conservation scores for selected variant	Table
6	Population differentiation	Estimation of population differentiation that measured by *F*_ST_	Heat map plot; Table
7	Natural selection	Natural selection signals for selected variant and/or its surrounding region	Manhattan plot, Table
8	Genomic diversity	Genomic diversity pattern for regions near selected variant	Scatter plot, Table
9	Linkage disequilibrium (LD)	Genetic linkage pattern for regions near selected variant	LD decay plot; Table

### High-performance database construction and user-friendly interfaces

**Fig. 3:**
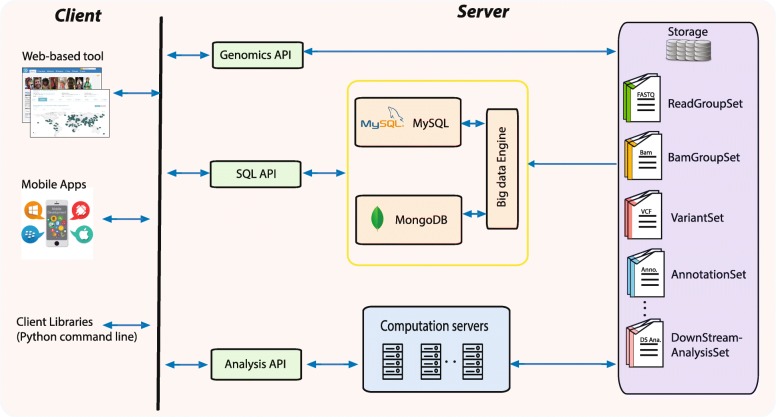
Construction of *PGG*.SNV database. SQL, Structured Query Language; API, Application Programming Interface; App, Application

Genomics is a big data science [93], and one of its biggest challenges is the extreme variety of data and an even greater variety of file formats [94]. *PGG*.SNV applies different strategies for storing, processing, exploring, and/or querying the diverse data types that have been generated, collected, analyzed, and annotated (Fig. 3). Upstream data, such as short read files and .bam files, have been deposited in a Huawei data storage system, which has at least 500 Terabyte dedicated for use by *PGG*.SNV. For small downstream data, such as sample and population information, the data are imported into a MySQL database (with 10 Terabyte storage volumes) as relationship data. For larger downstream data, such as annotations, we imported them into MongoDB clusters currently comprised of three servers, each of which has 12 Terabyte local storage and at least 64 Gigabyte memory. The largest collections in the *PGG*.SNV MongoDB database documents the counts of genotypes for each SNV in each population and contain 10 billion items at the time of the first release of *PGG*.SNV. For downstream analyses (such as linkage disequilibrium and genomic diversity analysis) that can generate extreme large data sets, *PGG*.SNV does not store the data but instead performs the corresponding analysis in real-time using genomic application programming interfaces (genomic API) (Fig. 3).

**Fig. 4:**
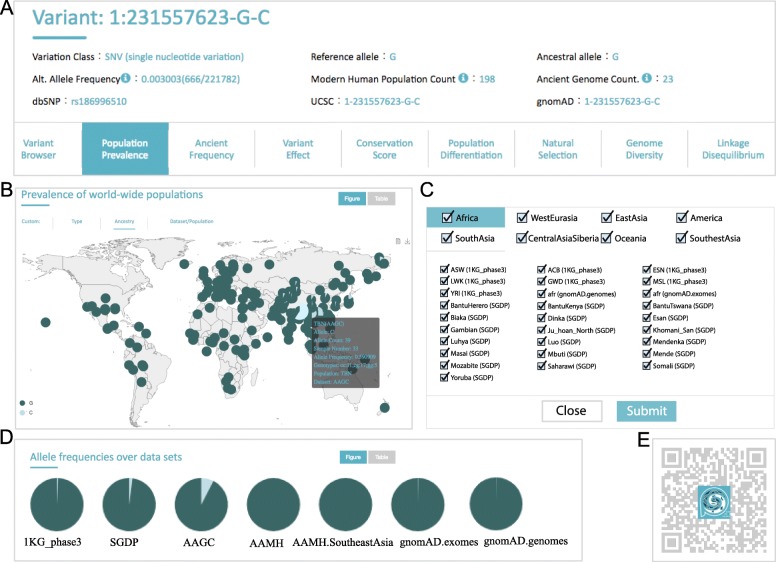
An example of the user-friendly method for visualization and accessing data. **a** Basic information for the selected SNV. Alt. Allele Frequency denotes the frequency of alternative alleles in the *PGG*.SNV database, with the alternative allele counts and total allele counts shown in brackets. The Modern Human Population Count represents the number of ethnic groups whose genomic data contain the selected variant in the *PGG*.SNV database. The Ancient Genome Count denotes the number of ancient genomic data sets that contain the selected variant in the *PGG*.SNV database. At the bottom of **a**, there are nine annotation cards for a selected variant. Users can switch them to visualize the corresponding annotation. **b** Allele frequencies of the variant across worldwide populations. The figure is interactive on the web, with an allele frequency pie chart of each population in a worldwide map where geographic locations represent the position of slices for corresponding populations. It has embedded mouse-scrolling events allowing the user to zoom in and out the resolution, a mouse-hovering event on a slice to get detailed information, and a figure- and table-switching event. **c** Custom pop-up windows for selecting populations, ancestries, and data sets. Note that the choices between population, ancestry, and data set buttons are related but not independent. **d** Allele frequencies of a variant in different data sets. **e** WeChat Quick Response (QR) code for access to the information including that in the *PGG*.SNV database. Users can scan the code and follow the PGGbase official account to access data via a smart phone

*PGG*.SNV provides three types of approaches to access data. A web-based service is the major, more user-friendly method, as it supports not only data accessibility but also result visualization. By searching genetic variants by physical position or RSID, *PGG*.SNV currently returns nine annotations (as mentioned in the above section) for the corresponding variant if it has been included in the database. By querying by a genomic region, official gene symbol or Ensembl gene name, the website returns all of the variants that meet the requirement and users can further select one for which to visualize the annotation web. Each type of annotation map comes with one or more figures or tables with interactive website elements (such as mouse hover and wheel scroll events) to illustrate the result (Fig. 4a and Additional file 3: Table S2). For instance, in the population prevalence annotation section for rs186996510, which is an adaptive variant in Tibetan highlanders [28–33], *PGG*.SNV initially returns an interactive figure (Fig. 4b), plotting the allele frequency pie charts of each population in a worldwide map where geographic locations represent the position of slices for the corresponding populations. By hovering the mouse on each slice, users can get detailed information such as the population name, ancestry, and sample size (text in shaded box of Fig. 4b) for the population denoted by that slice. By scrolling the mouse wheel, users are able to zoom the resolution of the map in and out to focus on specific regions. Moreover, users can customize the specific data sets, ancestries, or populations to be shown in the returned results (Fig. 4c) and can switch the result pattern from figure to table (at the top right corner of Fig. 4b) to obtain results in a .txt file or other file formats. Beside the population prevalence, the web summarizes the prevalence pattern of a derived allele in the assigned data sets (Fig. 4d). This function distinguishes the derived allele frequency differentiation between various data sets, facilitating the understanding of data set bias in the analysis of an allele’s prevalence. More specifically, in Fig. 4d, the derived allele frequency of 1:231557623-G-C (G allele) was higher in the AAGC data set (7.6%) than in other data sets (< 2.0%), because AAGC includes more genomes of East Asian populations, especially Tibetan highlanders. *PGG*.SNV has also embedded a web-based tool (https://www.pggsnv.org/tools.html) for the generation of figures after users have uploaded their own analyses.

In addition to the web-based interface, users can query variants using a mobile application (App) by linking to the WeChat official account named PGGbase (Fig. 4e and Additional file 2: Figure S2). WeChat can then return corresponding results (currently population prevalence) from *PGG*.SNV. Lastly, *PGG*.SNV offers client libraries (using python run on the command line) that run on a user’s own platform to query result in batch (Fig. 3), so that developers (currently restricted to collaborators) can incorporate these libraries into their own unique bioinformatics analysis pipelines.

### Prevalence of Mendelian-inherited disease variants across populations

**Fig. 5:**
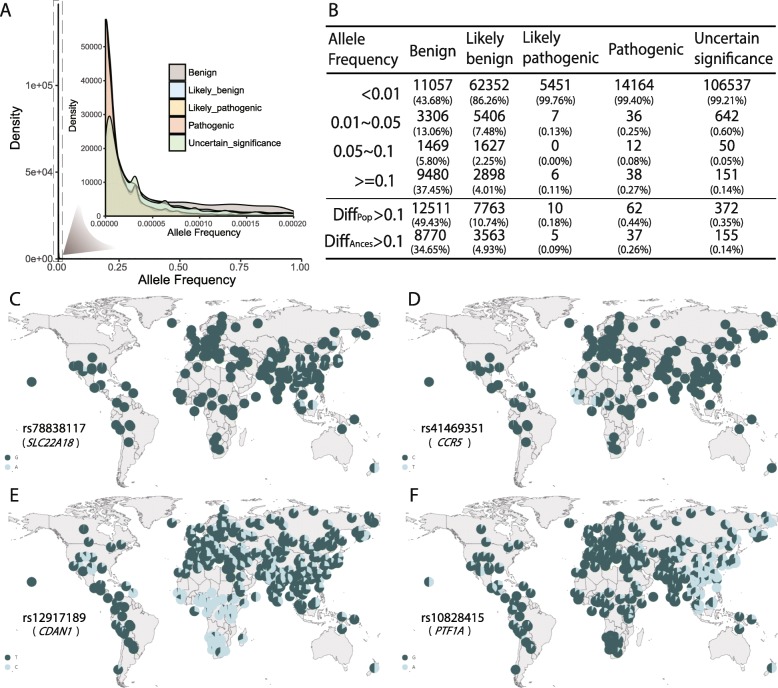
Prevalence and differentiation of Mendelian disease variants across populations. **a** Allele frequency spectrum of Mendelian-inherited disease variants. Mutations are grouped into five categories by their severity (see “Population and ancestry assignment”). **b** Rareness and population and ancestry differentiation of Mendelian-inherited disease variants. **c** An example of a pathogenic variant (rs78838117) that shows high DAF in some Southeast populations such as Bateq people (DAF = 0.33), Jakun people (DAF = 0.15), and Mendriq people (DAF = 0.125) in Malaysia. The corresponding *PGG*.SNV link is https://www.pggsnv.org/searchinfo.html?key=11-2930440-G-A. **d** An example of a pathogenic variant (rs41469351) that shows high DAF in some West Africa populations such as the Gambian people (DAF = 0.36). The corresponding *PGG*.SNV link is https://www.pggsnv.org/searchinfo.html?key=3-46412262-C-T. **e** An example of a variant (rs12917189) that shows large differentiation between Africans and non-Africans. The corresponding *PGG*.SNV link is https://www.pggsnv.org/searchinfo.html?key=15-43023482-T-C. **f** An example of a variant (rs10828415) that shows large differentiation between East Asian and other ancestral populations. The corresponding *PGG*.SNV link is https://www.pggsnv.org/searchinfo.html?key=10-23482850-G-A

*PGG*.SNV contains a substantial number of genetic variants from diverse populations of different ancestries, providing an extraordinary platform for dissecting the rareness of Mendelian-inherited disease variants in humans as a whole and for assessing their prevalence in diverse ethnic groups. We therefore systematically estimated the population prevalence of Mendelian-inherited disease-associated alleles (hereafter referred to as DAAs) based on *PGG*.SNV. We found that although most of DAAs in ClinVar are rare (Fig. 5a), 7.0% of these variants had a frequency of causal alleles larger than 0.05 in humans based on the allele frequency spectrum of all genomes that collected by *PGG*.SNV (Fig. 5b and Additional file 3: Table S2). This probably indicates that the phenotypes caused by these variants have had little effect on fitness during human evolutionary history. The proportions vary in five different variant groups, with 0.35% being pathogenic variants, 0.10% likely pathogenic, 0.19% uncertain significance, 6.26% likely benign, and 43.2% benign variants (Fig. 5b and Additional file 3: Table S2), suggesting that the more severe the variant group, the rarer the causal alleles. This result is expected since the classification of severity of Mendelian disease-related variants by the American College of Medical Genetics and Genomics (ACMG) [91] partly relied on allele rareness or population data obtained from Exome Sequencing Project, 1000 Genomes Project, or Exome Aggregation Consortium.

### Population differentiation of Mendelian-inherited disease variants

Mendelian-inherited disease-associated alleles are expected to be rare or in low frequency. As mentioned above, we did observe that many Mendelian disease variants often have high allele frequencies in different populations, suggesting that a Mendelian-inherited disease-associated allele defined or identified in one study or population could be a benign one in other populations, it is also true vice versa. We therefore investigated whether the frequencies of DAA differed between populations or ancestries. We identified a substantial number of 72 very severe variants (62 pathogenic variants and 10 likely pathogenic variants) and 20,274 less-severe variants (12,511 benign variants and 7763 likely benign variants) that show differentiation between ethnic groups (Diff_Pop_ > 0.1) (Fig. 5b and Additional file 4: Table S3). Some distinguished examples are rs78838117 in the gene *SLC22A18* (MIM: 602631), rs41469351 in the gene *CCR5* (MIM: 601373), rs1024196 in the gene *DST* (MIM:113810), and rs150877473 in *EPAS1* (MIM: 603349). rs78838117 is a pathogenic variant in ClinVar and is associated with rhabdomyosarcoma. Its derived allele frequency (DAF) is high in some Southeast Asian populations such Bateq people (DAF = 0.33), Jakun people (DAF = 0.15), and Mendriq people (DAF = 0.125) in Malaysia, while the average DAF of worldwide populations is as low as 0.01 (Fig. 5c). rs41469351 is another pathogenic variant which is associated with maternal transmission of human immunodeficiency virus, and the derived allele of rs41469351 is common in West African populations such as the Gambian people (DAF = 0.36) but is rare in non-African populations such as Han Chinese (DAF = 0) and West Eurasians (DAF = 0) (Fig. 5d). rs1024196 is related to hereditary sensory and autonomic neuropathy type IV, and the derived allele is enriched in some Africans, especially in the Xuun and Mbuti Pygmy populations. For instance, DAF is as high as 0.961 in Xuun and Mbuti Pygmy, while the mean DAF across all global ethnic groups is only 0.097. Another example is that rs150877473 contributes to familial erythrocytosis and shows an extremely high DAF in Tibetans (0.88), while the derived allele is nearly absent in non-Tibetan populations (0.021).

Meanwhile, we found 42 severer variants (5 pathogenic variants and 37 likely pathogenic variants), 12,333 functionally less-severe variants (8770 benign variants and 3563 likely benign variants), and 155 uncertain significant variants that show differentiation among populations of distinct ancestries (Diff_Ances_ > 0.1) (Fig. 5b and Additional file 5: Table S4). Remarkably, the DAF of rs12917189 was largely different between African (0.821) and non-African populations, such as South Asians (0.271), East Asian (0.175), West Eurasian (0.236), American (0.126), Southeast Asian (0.015), Central Asian and Siberian (0.214), and Oceanic populations (0.260) (Fig. 5e). rs12917189 is located in the gene *CDAN1 (MIM: 607465)*, and the derived allele C contributes to congenital dyserythropoietic anemia or congenital dyserythropoietic anemia, type I. This phenotype is more prevalent in Africa and is reported to play a role in resistance to malaria [95, 96]. Another example is rs10828415, which shows a large difference in the DAF comparing East Asians (0.387) and non-East Asian populations such as African (0.078), South Asian (0.050), West Eurasian (0.040), Americans (0.139), Southeast Asian (0.020), Central Asian and Siberian (0.114), and Oceanic populations (0.036) (Fig. 5f). The variant is located in the gene *PTF1A* (MIM: 607194) and can lead to permanent neonatal diabetes mellitus according to the ClinVar database.

The above results suggest that a large number of the Mendelian-inherited disease variants, while assumed to be rare in frequency including those pathogenic or deleterious, vary in populations and ancestries, reflecting that health disparities exist extensively in human populations and ancestries. This pattern is likely to be shaped by the complex demographic history as well as local adaptations experienced by early humans or their descendants after population divergence. Therefore, it is of the utmost importance to concentrate on diverse populations and families with different genetic backgrounds when mapping causal variants for Mendelian-inherited diseases. *PGG*.SNV provides such a platform for examining allele frequency and various population genetic parameters in several hundred diverse populations worldwide.

### Cautions for interpreting genetic variants

Recently, an increased number of researchers investigate the allele frequency of variants in human populations and predict functional impacts or causality for each variant according to an allele’s rareness in medical studies [7, 36–38]. Though there has been a dramatic increase in the number of genomes sequenced for diverse human populations, most population prevalence annotation tools [87, 97] are frequently based on a few number of data sets, especially on 1KGP [4] and gnomAD [39], which are absolutely valuable reference panels. However, these data sets are insufficient to cover the majority of ethnic groups and therefore are not able to represent comprehensive genomic diversity of human populations. Concentration on specific data sets or attempts to use estimates of genetic risk from unrelated ancestral populations in a population may introduce frequency bias at the population level, ancestry level, and data set level, and may result in inaccurate assessment. First, one common allele in one population of a specific ancestry may be rare in another population of different ancestries and vice versa. For example, rs10828415 is common in East Asian (DAF = 0.387) but rare in Southeast Asians (DAF = 0.020) and South Asians (DAF = 0.050), even though all of these populations are located in Asia (Fig. 5). Second, one common allele in a specific population may be rare in populations of the same ancestry and vice versa. For example, rs150877473 shows an extremely high DAF in Tibetan population (0.88) but an extremely low DAF in Han Chinese (0.03), even though these two populations are from the same ancestral population [34, 98]. Lastly, one common allele in a specific data set could be rare in another data set and vice versa. Consider that the derived allele in rs186996510 is rare in the 1KGP data set (0.0065) but relatively common in the AAGC data set (0.075). We therefore suggest that researchers or organizations that specify standards and guidelines for the interpretation of sequence variants should investigate a sufficient number of populations of different ancestries so as to decrease the bias or error rates in future studies. By generating and collecting the genomes of diverse data sets (including 1KGP and gnomAD) from various ancestries and populations, *PGG*.SNV provides an extraordinary tool for dissecting the implications of human SNVs.

According to the frequency spectrum of Mendelian-inherited disease variants (Fig. 5a, b), the causal alleles of many variants (7.0%) are not rare (< 5% is often the criteria for defining a variant as rare or common), indicating that the commonly used standard of assuming that causal variants should be rare when mapping causal variants may not be applicable to many of the Mendelian diseases. There are many factors that may change disease risk alleles from rare to common, including the following: (1) if a disease had little effect on fitness during human evolution or the age of the onset is very late, the allele frequency could be shifted by random genetic drift; (2) the prevalence of a disease allele could be elevated by positive selection if the allele was once advantageous during human evolution, a selective sweep on a deleterious mutation in *CPT1A* in arctic populations is such an example [25]; (3) genetic hitch-hiking during a selective sweep could increase the frequency of moderately deleterious mutations; and (4) strong bottlenecks in the history of a population would accumulate alleles associated with recessive disorders [22–24, 79]. We suggest that researchers should loosen the criteria of population prevalence when identifying causal alleles for Mendelian-inherited diseases or populations that may potentially meet the above conditions to avoid false negative results.

## Conclusion

*PGG*.SNV provides reference genomic resources for diverse human populations, particularly including those from indigenous Asian populations (Additional file 6). With a comprehensive catalogue of genetic variants and annotations, *PGG*.SNV enables studies of variants that are rare or not existing in well-studied populations, and provides the population prevalence of variants in various populations with little ancestral bias and further guides Mendelian-inherited disease mapping studies. *PGG*.SNV documents many ancient genomes and compares them with contemporary human genomes, allowing researchers to understand the evolutionary trajectory of genetic variants as well as gene flow or introgression events. Moreover, this database improves interpretations of putative causal loci for Mendelian diseases, population differentiation analysis, and adaptation to local environments for global populations. Eventually, *PGG*.SNV will help advance our understanding of the biological meaning of the human genome sequence in light of human evolution.

## Supplementary information

**Additional file 1: Table S1.** The list of populations included in PGG.SNV.

**Additional file 2: Figure S1.** Principal component analysis (PCA) for each data set in *PGG*.SNV. Figure S2. Steps for querying variant via WeChat.

**Additional file 3: Table S2.** The list of Mendelian-inherited disease variants with alternative allele frequency larger than 0.05 in PGG.SNV.

**Additional file 4: Table S3.** The list of Mendelian-inherited disease variants showing large differentiation between populations (Diff_pop_ > 0.1).

**Additional file 5: Table S4.** The list of Mendelian-inherited disease variants showing large differentiation between ancestries (Diff_Ances_ > 0.1).

**Additional file 6.** Review history.

## Data Availability

All the summary statistics of genomic variants on the population level can be freely downloaded from the *PGG*.SNV website (https://www.pggsnv.org/). No password or license is required to access the data provided on the database website. The individual genotype data are not allowed to be released to the public according to the local IRB’s policies. The list of all datasets can be seen in Table 1. The following lists the web resources mentioned in this paper: The 1000 Genomes, http://www.internationalgenome.org/ ClinVar, https://www.ncbi.nlm.nih.gov/clinvar/ gnomAD Browser, http://gnomad.broadinstitute.org/ *PGG*.Population, https://www.pggpopulation.org MyVariant.info, http://myvariant.info/ Variant Effect Predictor, https://asia.ensembl.org/info/docs/tools/vep/index.html Picard, http://broadinstitute.github.io/picard/ Estonian Biocentre, http://evolbio.ut.ee/
