# Supplementary material for: PGG.SNV: understanding the evolutionary and medical implications of human single nucleotide variations in diverse populations
Source: Genome Biol. 2019 Oct 22;20:215. doi: 10.1186/s13059-019-1838-5 (PMC6805450; doi:10.1186/s13059-019-1838-5)
Supplement: Supplementary file 2 — Additional file 2: Figure S1. Principal component analysis (PCA) for each data set in PGG.SNV. Figure S2. Steps for querying variant via WeChat. [file 13059_2019_1838_MOESM2_ESM.pdf]

Supplemental Information for

PGG.SNV: Understanding the evolutionary and medical implications of human single nucleotide variations in diverse populations

Chao Zhang, Yang Gao, Zhilin Ning, Yan Lu, Xiaoxi Zhang, Jiaojiao Liu, Bo Xie, Zhe Xue, Xiaoji Wang, Kai Yuan, Xueling Ge, Yuwen Pan, Chang Liu, Lei Tian, Yuchen Wang, Dongsheng Lu, Boon-Peng Hoh, Shuhua Xu.

* Correspondence to: xushua@picb.ac.cn

This PDF file includes:
Supplemental Methods
Supplemental References
Supplemental Figure S1 and S2

Supplemental Methods

1. DNA Sample Preparation and Sequencing of Asian Admixed Genomes Consortium (AAGC)

Genomic DNA of 1009 individuals was extracted from the blood samples using QIAGEN DNeasy Blood & Tissue Kit. DNA concentrations were measured with the NanoDrop 2000 (Thermo Fisher Scientific), and sheared with Covaris S220 Sonicator (Covaris) to target of 500 - 600 base pairs (bp) average size. Fragmented DNA was purified using Sample Purification Beads (Illumina). Adapter-ligated libraries were prepared with the TruSeq Nano DNA Sample Prep Kits (Illumina) according to Illumina-provided protocol. DNA concentrations of the resulting sequencing libraries were measured with the Qubit 2.0 fluorometer dsDNA HS Assay (Thermo Fisher Scientific). Quantities and sizes of the resulting sequencing libraries were analyzed using Agilent BioAnalyzer 2100 (Agilent). The libraries were used in cluster formation on an Illumina cBOT cluster generation system with HiSeq X HD PE Cluster Kits (illumina). Whole-genome sequencing, with a target coverage 10-30× for 150 bp paired-end reads, was performed in WuXi NextCODE at Shanghai using an Illumina HiSeq X following Illumina-provided protocols. Each sample was run on a unique lane with at least 90 GB PF data and the quality of the reads data were controlled for ensuring that 80% of the bases achieved at least a base quality score of 30.

2. Alignment, variant identification and filtering for AAGC data set

Reads mapping

Per-individual sequence reads were aligned using 'mem' algorithm "bwa mem –M –R @RG\tID:name\tSM:name" in the Burrows-Wheeler Algorithm (BWA) version 0.7.10-r789 [1] to the reference human genome (GRCh37), and then converted to BAM format, sorted by genomic position and indexed using samtools version 0.1.19-44428 [2]. To make full use of our computational resources in parallel in the downstream sequence data processing steps, we filtered out the reads with MAPQ < 20 using 'samtools view –q20' and split the single BAM file according to chromosomes.

Duplicate marking

Picard toolkit version 1.117 was used to mark the potential duplicate reads inherited from library construction step, in which the amplified PCR errors can introduce the wrong variants in variants calling [3,4]. The MarkDuplicates.jar in Picard was used for chromosome-wise duplicates marking per-individual.

Local realignment and base quality recalibration

Alignments in the combined BAM file were then locally realigned around known insertions/deletions (INDELs) using INDELs reported in the 1000 Genomes Project (KGP) Phase I as the training dataset. Base quality score were recalibrated to reduce

the base quality score bias from the sequencer, using INDELs and dbSNP (version 147) reported in KGP Phase I as the training data sets.

Variants calling

HaplotypeCaller module in GATK version 3.2-0-g289df4b [3,4] was used for SNPs and INDELs calling chromosome-wise simultaneously for each sample, as it is more accurate to call variants in some special region with *de novo* local assembly method, especially in calling INDELs. For population-based analyses, GATK GenotypeGVCFs module was applied to the GVCFs generated in the previous step to call the variants for each chromosome.

Variant qulity score recalibration and SNP filtering

The chromosome-wise raw variants were combined to genome-wide raw variants for population-based variants VCFs and individual-based GVCFs. GATK variants quality score recalibration (VQSR) module was used to filter the population based raw SNPs and INDELs, separately. Briefly speaking, VQSR used maximized sensitivity on these variants first, and then used some variants collections as training dataset to estimate the levels of specificity to filter these raw variants. For SNPs filtering, the variants collections contained HapMap 3.3 genotyping result, OMNI genotyping dataset, KGP Phase I high confident SNPs and dbSNP dataset. For INDELs filtering, the variants collections contained Mills and KGP Phase I gold standard INDELs. After VQSR, we filtered variants with the universal masks including regions with (a) low mappablity mask similar with Li and Durbin [5]; (b) low complexity mask encompring regions made by mDUST, and homopolymers and repeat regions obtained from UCSC.

Supplemental References

1. Li, H. & Durbin, R. Fast and accurate long-read alignment with Burrows – Wheeler transform. *Bioinformatics* **26,** 589–595 (2010).
2. Li, H. *et al.* The Sequence Alignment / Map format and SAMtools. *Bioinformatics* **25,** 2078–2079 (2009).
3. Mckenna, A. *et al.* The Genome Analysis Toolkit : A MapReduce framework for analyzing next-generation DNA sequencing data. *Genome Res.* **20,** 1297–1303 (2010).
4. Depristo, M. A. *et al.* A framework for variation discovery and genotyping using next-generation DNA sequencing data. *Nat. Genet.* **43,** 491–498 (2011).
5. Li, H. & Durbin, R. Inference of human population history from individual whole-genome sequences. *Nature* **475,** 493–496 (2011).

Supplemental Figures

Figure S1

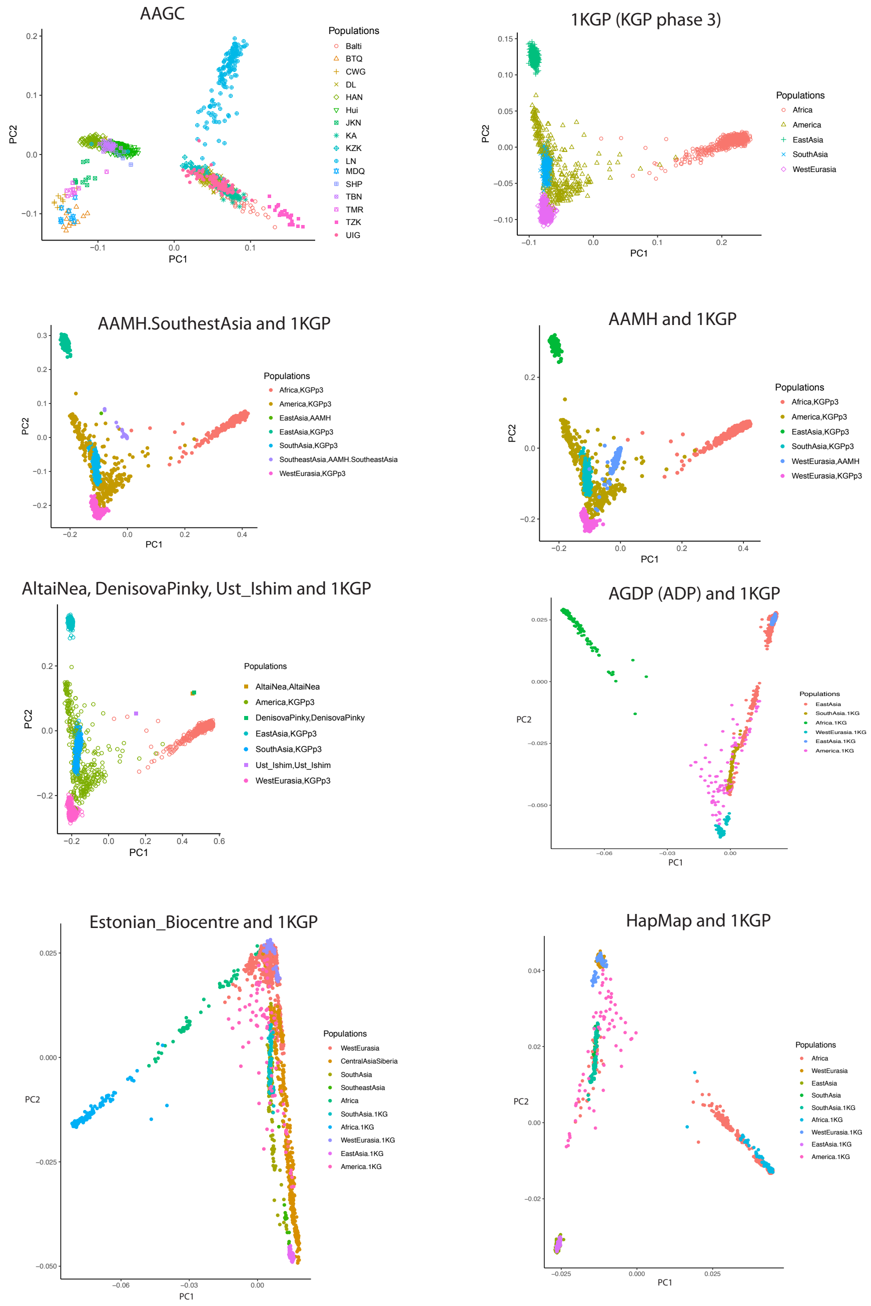

Figure S1 (Cont.)

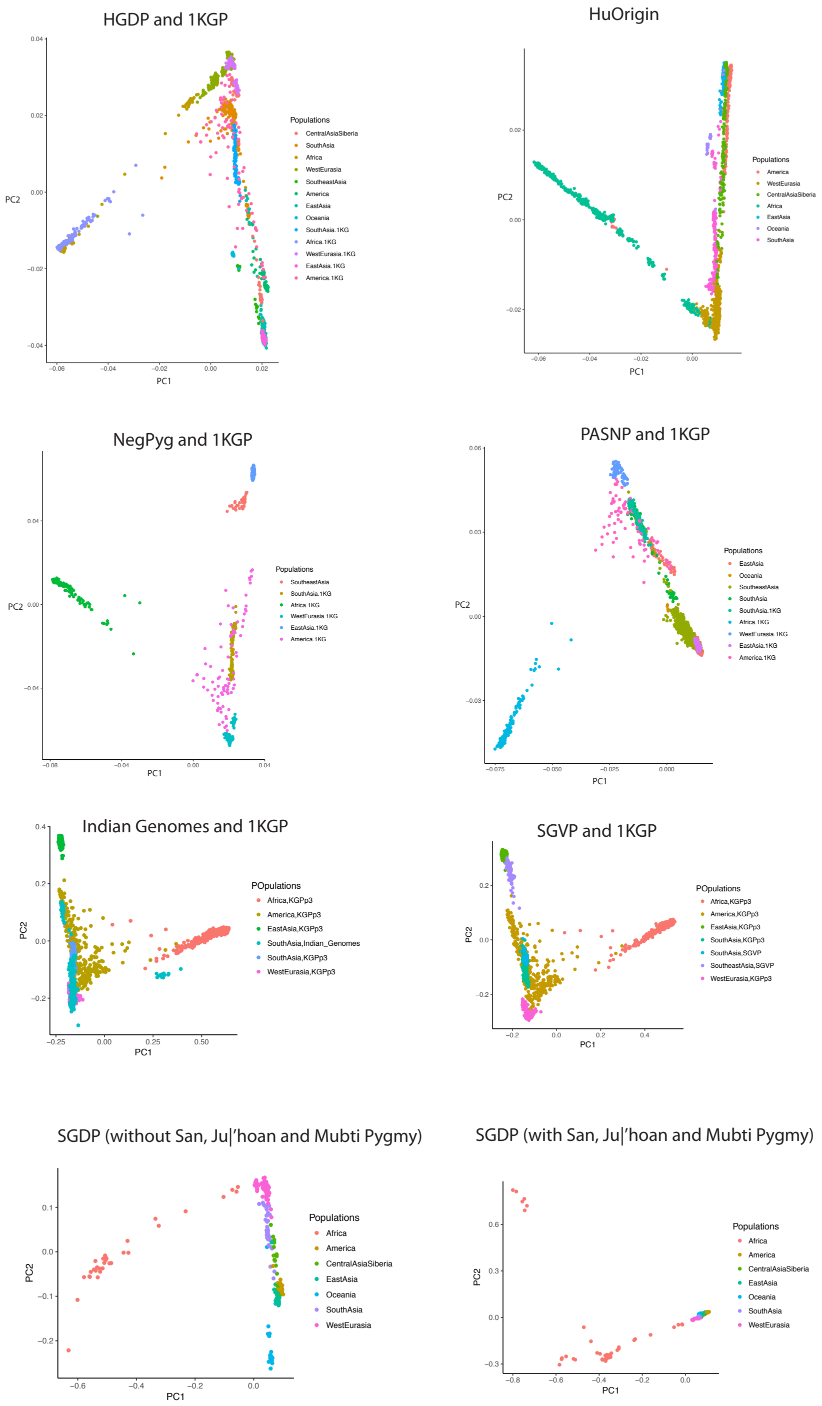

Figure S1. Principal component analysis (PCA) for each data set in *PGG*.SNV. Note that only data sets with genotypes but not only frequency information were performed by PCA.

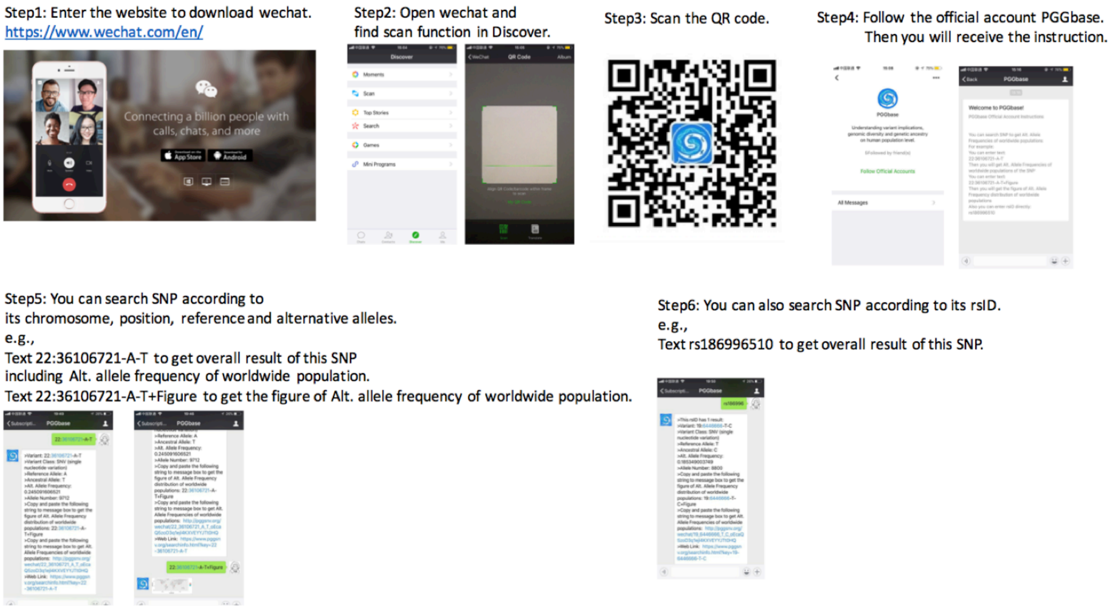

Figure S2. Steps for querying variant via WeChat.
