# Supplementary material for: PGG.SNV: understanding the evolutionary and medical implications of human single nucleotide variations in diverse populations
Source: Genome Biol. 2019 Oct 22;20:215. doi: 10.1186/s13059-019-1838-5 (PMC6805450; doi:10.1186/s13059-019-1838-5)
Supplement: Supplementary file 6 — Additional file 6. Review history. [file 13059_2019_1838_MOESM6_ESM.docx]

Review History

**First round of review**

**Reviewer 1**

Zhang et al. described a database of human SNVs called PGG.SNV. The significance and uniqueness of this database is that it's the most comprehensive and diversified SNV-centric resources as to the populations represented. Especially, it included 16 populations from the Asian Admixed Genomes Consortium and 38 populations from the Han Chinese Genomes Project, which are new and not published. Along with other Asian populations collected in the database, the database is a good complementary as to other SNV frequency databases that are very much European focused. It also includes quite a large number of ancient human genome resources, which can be also very useful. As to the SNVs, some basic but useful summary statistics are provided including population prevalence, ancient frequency, population differentiation, natural selection, linkage disequilibrium, etc., all of which are important information related to the function of the SNVs. 

However, I also find that some critical information is missing, without which it is hard to make a judgement for the manuscript as a whole.

1. It does not have a list of populations collected in the database. Since it is long, it can be in the supplementary file. Without such a list, I don't know what the 16 populations from the Asian Admixed Genomes Consortium and 38 populations from the Han Chinese Genomes Project really are, for example.

2. In "Prevalence of Mendelian-inherited disease variants across populations", they reported 6.7% of the variants had a frequency of causal alleles larger than 0.05. This result is interesting and potentially important, but the authors do not provide a list of those SNVs.

3. In "Population differentiation of Mendelian-inherited disease variants", the authors reported 65 very severe variants that show Diffpop>0.1, and 36 severer variants with Diffances >0.1, but again I cannot find the list of those SNVs.

4. They did not explain why 0.1 was used as a cut-off for variants with significant difference between populations or ancestries.

Some minor issues:

1. The background part is too long and not very focused. It also lacks a comparison of PGG.SNV with existing similar databases and resources, including their own PGG.population.

2. what's the meaning of the right-most hexagonal in figure 1?

**Reviewer 2**

The study "PGG.SNV: Understanding the evolutionary and medical implications of human single nucleotide variations in diverse populations" describes a population genetic resource combining ancient genomes with East and South Asian genomes. While the resource and study address a very timely topic in human genetics: population diversity, there are several major issues that should be addressed. 

Major comments: 

1. It is not clear why gnomAD exomes and genomes not used for this study, as they contain numerous East and South Asian genomes. Mentioning in page 5 that the ethnic groups in gnomAD are unknown and therefore allele frequencies from gnomAD should be ignored is misleading: gnomAD use accurate PCA predictions to predict the major population groups in resource (although not in high resolution for some populations). 

2. Population assignment is not clear. It seems that it was done only based on geography. However, it is important to assign genetic ancestry by PCA which is significantly more accurate than geographic origin of sampling and/or self-reporting.

3. It is highly recommended using the updated GRCh38 reference genome instead of previous versions.

4. Regarding the varying results across different populations: the authors should test whether some are due to alignment issues resulting in incorrect variant calling, where the current human reference genome (mostly European) is insufficient for these populations.

5. The manuscript requires a substantial English editing.

6. Export function on the webpage doesn't work.

7. The webpage would much benefit from added flexibility due to the large number of populations, such as allowing choice of population groups to display, different damage prediction scores etc., where the gnomAD template (open source) could potentially be used.

Minor comments:  

1. The title and text mention "diverse populations", while the study focuses on East and South Asians. This should be explicitly mentioned. 

2. NGS (in particular exome and genome sequencing) is not mentioned in the Background section.

3. Mentioning positively selected regions in the context of pathogenicity is counter intuitive, as pathogenic mutations are more likely to be in regions under purifying selection.

4. The resource should be carefully compared with gnomAD and it should be better discussed why it is complementary.

**Authors Response**

**Point-by-point responses to the reviewers’ comments:**

Response to Reviewer #1 

Zhang et al. described a database of human SNVs called PGG.SNV. The significance and uniqueness of this database is that it's the most comprehensive and diversified SNV-centric resources as to the populations represented. Especially, it included 16 populations from the Asian Admixed Genomes Consortium and 38 populations from the Han Chinese Genomes Project, which are new and not published. Along with other Asian populations collected in the database, the database is a good complementary as to other SNV frequency databases that are very much European focused. It also includes quite a large number of ancient human genome resources, which can be also very useful. As to the SNVs, some basic but useful summary statistics are provided including population prevalence, ancient frequency, population differentiation, natural selection, linkage disequilibrium, etc., all of which are important information related to the function of the SNVs.

However, I also find that some critical information is missing, without which it is hard to make a judgement for the manuscript as a whole.

1. It does not have a list of populations collected in the database. Since it is long, it can be in the supplementary file. Without such a list, I don't know what the 16 populations from the Asian Admixed Genomes Consortium and 38 populations from the Han Chinese Genomes Project really are, for example.

*Response: Thank the reviewer for reminding us of this. In our revised manuscript, we provide the full list of populations in Table S1 as well as on the user guide page of our database (https://www.pggsnv.org/userguide.html).* 

2. In "Prevalence of Mendelian-inherited disease variants across populations", they reported 6.7% of the variants had a frequency of causal alleles larger than 0.05. This result is interesting and potentially important, but the authors do not provide a list of those SNVs.

*Response: Following the reviewer’s suggestion, we provided the list of disease variants with causal alleles larger than 0.05 (See Table S2).* 

3. In "Population differentiation of Mendelian-inherited disease variants", the authors reported 65 very severe variants that show Diffpop>0.1, and 36 severer variants with Diffances >0.1, but again I cannot find the list of those SNVs. 

*Response: In our resubmitted manuscript, all severer variants with Diffpop>0.1 and Diffances >0.1 can be obtained in Table S3 and Table S4, respectively. We thank the reviewer for reminding us, and we have already provided all the aforementioned lists.* 

4. They did not explain why 0.1 was used as a cut-off for variants with significant difference between populations or ancestries. 

*Response: We understand the reviewer’s concern. We also believe that the cut-off for variants with significant difference should be defined based on the tail of the distribution for the differentiation pattern of the whole genome variants. However, it would be very complicated and infeasible to apply different cut-offs as the database archive very huge number of populations, and the populations are diverse and in a hierarchical structure among data sets. Since we focus on Mendelian disease variants which may have severe effects, they are in relatively low frequency in human, and are usually not highly-differentiated among populations. According the allele frequency distribution of disease variants in PGG.SNV (Figure 5A and 5B), 0.1 is close to the top 5% cut-off value for most of the populations. We therefore expect that the difference between populations or ancestries with 0.1 would be a significant difference for Mendelian variants. We have provided explanations in the revised manuscript (lines 1-3, page 11).* 

Some minor issues: 
1. The background part is too long and not very focused. It also lacks a comparison of PGG.SNV with existing similar databases and resources, including their own PGG.population. 

*Response: We tried to shorten the introduction in the revised version. We also compared PGG.SNV with other two frequently used data sets, 1KGP and gnomAD, in the resubmitted manuscript (lines 2-15, page 5; lines 8-20, page 12). We should clarify that the application/function of PGG.SNV is totally different from that of PGG.population. PGG.population was designed mainly for visualization of ancestry make-up of diverse populations, which focuses on “populations”, while PGG.SNV focuses on “variants”.* 

2. What's the meaning of the right-most hexagonal in figure 1? 

*Response: The ellipsis in the right hexagonal represents other population genomics analyses that are not included in the current version of the database but would be performed in later versions. In our revised manuscript, we specified the meaning of the hexagonal in the legend of figure 1 (line 3-5, page 31).* 

 
Response to Reviewer #2 

The study "PGG.SNV: Understanding the evolutionary and medical implications of human single nucleotide variations in diverse populations" describes a population genetic resource combining ancient genomes with East and South Asian genomes. While the resource and study address a very timely topic in human genetics: population diversity, there are several major issues that should be addressed. 

Major comments: 
1. It is not clear why gnomAD exomes and genomes not used for this study, as they contain numerous East and South Asian genomes. Mentioning in page 5 that the ethnic groups in gnomAD are unknown and therefore allele frequencies from gnomAD should be ignored is misleading: gnomAD use accurate PCA predictions to predict the major population groups in resource (although not in high resolution for some populations). 

*Response: We would like to clarify and confirm that we actually have already included the data from the gnomAD in this study (see Table 1 and S1). However, as the reviewer mentioned, gnomAD use PCA predictions to predict the major population groups in resource, but not in high resolution for some* *populations. In fact, the genomes in gnomAD were merely divided into 15 groups on continental level, leaving the majority of the specific ethnic groups unknown. For example, gnomAD exomes grouped East Asians roughly into three categories: “Korean”, “Japanese” and “other East Asians”. Therefore, users are not able to query the allele frequencies for most of East Asian populations, such as the Han Chinese, Tibetan and Uyghur populations and so on. That was why we interpret in page 5 that “the precise ethnic groups covered in the gnomAD project are unknown, so data from this should ignore variant frequencies for specific populations”. To clarify our point, we have revised the sentence in our resubmitted manuscript (lines 2-15, page 5). Thank the reviewer for pointing this out.* 

2. Population assignment is not clear. It seems that it was done only based on geography. However, it is important to assign genetic ancestry by PCA which is significantly more accurate than geographic origin of sampling and/or self-reporting. 

*Response: We totally agree with the reviewer that genetic ancestry is more accurate than geography in grouping genomes. We would like to clarify that population assignment in our database was done majorly based on genetic ancestry but not only on geography and other factors. The details are as follows: 
(1) When a dataset is generated or collected, the genetic affinity and population structure were firstly assigned based on PCA. Population and/or sample outliers that conflicting the geographic origin of sampling and/or self-reporting would be excluded in our database. For example, the figure below shows the genetic architecture of the populations for the AAGC dataset. And PCAs for each dataset are now provided in Figure S1 in our revised manuscript. 
(2) For a population with extremely large sample size and clear sampling locations, we then divided it into different subgroups. In the current version of PGG.SNV, the Han Chinese from Han Chinese Genomes Project (n = 51,094) is the only case, as it is the world’s largest ethnic group and previous studies have shown their sub-structures (Xu et al., AJHG 2009; Chen et al., AJHG 2009; Chiang et al., MBE 2018). 
(3) For ancient human genomes, we assigned the population based on geography (country) and their time periods, as we don’t know which exactly ethnic group they belong to, though we may infer their rough ancestry. Moreover, the time- and country-based population assignment for ancient genomes facilitate users to trace the allele frequency fluctuation through history and thus to understand the genetic origin of a specific variant. 
To make the population assignment process more clear, we revised the corresponding section in our resubmitted manuscript (lines 14-26, page 9).*

3. It is highly recommended using the updated GRCh38 reference genome instead of previous versions. 

*Response: Following the reviewer’s suggestion, we have made a lot of efforts and updated the genetic variants to GRCh38 assembly (https://grch38.pggsnv.org/). For convenience, we also kept the genome build GRCh37 (https://www.pggsnv.org/). Users can easily switch the builds though the drop-down menu (see the figure blow).* 

4. Regarding the varying results across different populations: the authors should test whether some are due to alignment issues resulting in incorrect variant calling, where the current human reference genome (mostly European) is insufficient for these populations. 

*Response: We highly appreciate the reviewer’s insightful comment. However, to be honest, it is challenging to estimate the incorrect variant calling induced by alignment due to reference genome bias. The first thing that comes to our mind is constructing a new reference genome for each population via de novo assembly. The work need to be better done with long reads sequenced by third-generation sequencing combined with high-coverage NGS, the cost is beyond the capacity of this study for this time. We once conceived the projects that assembling reference genomes for each of the East Asian populations, but this would be a long journey and would be considered in the future. 
In the current version of PGG.SNV, we applied the BWA and GATK best practices workflows, which is the most commonly used pipeline to our knowledge, to do alignment and call variants. All reads with low mapping quality were removed during our analysis. Only variants passed the quality score are included in the database. Users can obtain the quality for specific variant in each population from the database (see the red box in the figure below).* 

5.The manuscript requires a substantial English editing. 

*Response: We have carefully checked the language of the text, and we also used a commercial service for language editing provided by LetPub (www.LetPub.com). We hope the revised version has been significantly improved.* 

6. Export function on the webpage doesn't work. 

*Response: Thank the reviewer for reminding us of this issue. We have fixed the bug. Now the export function supports the most popular browsers (including Chrome, Safari, UC, Opera and Edge) which have more than 85% worldwide usage share (https://en.wikipedia.org/wiki/Usage_share_of_web_browsers).* 

7. The webpage would much benefit from added flexibility due to the large number of populations, such as allowing choice of population groups to display, different damage prediction scores etc., where the gnomAD template (open source) could potentially be used. 

*Response: We thank the reviewer for this suggestion and we agree that there is still space to improve the flexibility of the webpage. We tried to use the gnomAD template but it turned out the workload was beyond what we could complete in a few weeks. Therefore, we attempted to improve the flexibility of the webpage based on the current design. For example, we have provided different types of buttons to selected population groups to display in population prevalence page: (1) Through the “Ancestry” button, users can select populations from the ancestries that they select; (2) By clicking the “Dataset/Population” button, users can custom populations from different dataset or just select specific populations within one data set; (3) The “Type” button enable users to choose present-day populations and ancient populations if PGG.SNV includes both types of genomes for that variant. For the damage prediction scores, our database currently includes SIFT, PolyPhen2, phastCons, CADD and GERP scores. We will keep on annotating variants with other conservation scores and make the function more flexible.* 


Minor comments: 

1. The title and text mention "diverse populations", while the study focuses on East and South Asians. This should be explicitly mentioned. 

*Response: We thank the reviewer’s suggestion. We do agree with the reviewer that the genomes in our database represent more comprehensive populations in East Asia and Southeast Asia. In the abstract and conclusion section, we made it clear the number of newly-sequenced genomes and clarified that our database gives much higher weight to previously under-investigated indigenous populations in East and South Asia (line 5-6, page 2; line 7, page 20). As a matter of fact, despite we included more East Asian samples than other existing databases such as gnomAD, the number of European genomes (n = 90053, Fig. 2A) is still larger than that of East Asian samples (n = 60717, Fig. 2A) in our PGG.SNV database. Therefore, our database is not only focusing on East and South Asians but also on other populations (977 populations or ethnic groups in total).* 

2. NGS (in particular exome and genome sequencing) is not mentioned in the Background section. 

*Response: Following the suggestion of the reviewer, we have provided some description for NGS (line 3, page 3).* 

3. Mentioning positively selected regions in the context of pathogenicity is counter intuitive, as pathogenic mutations are more likely to be in regions under purifying selection. 

*Response: We totally agree with the reviewer that pathogenic mutations are more like to be under purifying selection. The point of view can be verified by the allele frequency distribution of Mendelian disease variants in PGG.SNV (Figure 5A): the majority of disease alleles (93%) are in low frequencies (derived< 0.05). However, in some cases, deleterious allele could be elevated by positive selection if the allele was once advantageous during human evolution. A selective sweep on a deleterious mutation (rs80356779) in CPT1A in arctic populations is such an example. A previous study showed that the selective sweep has driven this variant to high frequency in circum-Arctic populations within the last 6–23 ka despite associated deleterious consequences, possibly as a result of the selective advantage it originally provided to either a high-fat diet or a cold environment (Clemente et al., AJHG 2014). Below, we also provided the allele frequency of the deleterious allele A in worldwide populations in PGG.SNV database. 
Thanks the reviewer for reminding us of the counter intuitive issue. To clarify our point view, we provided rs80356779 in CPT1A as an outstanding example in our revised manuscript (lines 25-26, page 19).* 

4. The resource should be carefully compared with gnomAD and it should be better discussed why it is complementary. 

*Response: Following this suggestion, we have further compared PGG.SNV with gnomAD (lines 2-15, page 5), discussed the difference of our database from the gnomAD (lines 9-20, page 12), and explained why it is complementary (lines 18-25, page 18; lines 13-14, page 19). 


Reference 
Chiang CWK, Mangul S, Robles C, Sankararaman S: A Comprehensive Map of Genetic Variation in the World's Largest Ethnic Group-Han Chinese. Mol Biol Evol 2018, 35:2736-2750. 
Xu S, Yin X, Li S, Jin W, Lou H, Yang L, Gong X, Wang H, Shen Y, Pan X, et al: Genomic dissection of population substructure of Han Chinese and its implication in association studies. Am J Hum Genet 2009, 85:762-774. 
Chen J, Zheng H, Bei JX, Sun L, Jia WH, Li T, Zhang F, Seielstad M, Zeng YX, Zhang X, Liu J: Genetic structure of the Han Chinese population revealed by genome-wide SNP variation. Am J Hum Genet 2009, 85:775-785. 
Clemente FJ, Cardona A, Inchley CE, Peter BM, Jacobs G, Pagani L, Lawson DJ, Antao T, Vicente M, Mitt M, et al: A Selective Sweep on a Deleterious Mutation in CPT1A in Arctic Populations. Am J Hum Genet 2014, 95:584-589.*

**Second round of review**

**Reviewer 1**

I have no further comments.

**Reviewer 2**

The authors have addressed all my concerns.
